# The Role of Monocyte/Macrophage and CXCR3 in Differentiation between Recurrent Hepatitis C and Acute Cellular Rejection Postliver Transplantation

**DOI:** 10.1155/2018/2726939

**Published:** 2018-04-30

**Authors:** Asmaa Ibrahim Gomaa, Nermine Ahmed Ehsan, Ahmed A. Elrefaei, Mervat Mohamed Sultan, Maha Mohamed Elsabaawy

**Affiliations:** ^1^Department of Hepatology and Gastroenterology, National Liver Institute, Menoufia University, Shebin El Koum, Egypt; ^2^Department of Pathology, National Liver Institute, Menoufia University, Shebin El Koum, Egypt

## Abstract

**Objective:**

Liver transplantation (LT) is the recommended treatment for patients with advanced liver disease and cirrhosis in all guidelines, mostly as a complication of HCV. The distinction between reinfection of the graft with HCV and acute cellular rejection (ACR) is essential because they are managed differently. Hepatic macrophages, which can either arise from circulating blood-derived monocytes (BDM) or from resident tissue Kupffer cells, are central in the pathogenesis of chronic liver injury. The aim of this work was to evaluate whether the origin of macrophages and the immune mediator CXCR3 could help in differentiating between acute recurrent HCV and ACR after liver transplantation.

**Methods:**

Twenty-nine cases of recurrent hepatitis C and 26 cases of ACR were included in this study. The expression of CD 68 (macrophage marker), CD11b (BDM marker), and CxCR3 in the postliver transplant biopsy using immunohistochemistry was determined.

**Results:**

CD11b expression highlighting macrophages of BDM origin was in favor of recurrent hepatitis C (*P* < 0.001) than in ACR (*P* = 0.44), while CXCR3 expression by hepatocytes was in favor of ACR (*P* = 0.001).

**Conclusion:**

Macrophage infiltrating liver tissue post LT can distinguish between ACR by upregulation of CXCR3 and recurrent hepatitis C by predominant CD11b.

## 1. Introduction

Chronic hepatitis C virus infection (HCV) is a major cause of end-stage liver disease that has been increasingly the important indication for liver transplantation (LT) globally. HCV reinfection of the graft occurs almost universally, leading to graft injury in the majority of patients and cirrhosis in 8–44% in 5–7 years after reinfection [1]. The viral load may be influenced by corticosteroid intake [2], and the histologic features of recurrent hepatitis C may be modified by immunosuppressive therapy, which harden its differentiation from acute cellular rejection (ACR) [3]. Allograft failure is the most common cause of death and retransplantation among those recipients [4–7].

ACR is encountered in 18%–30% of transplanted patients leading to allograft failure [8, 9]. The differential diagnosis between recurrent HCV and ACR is often difficult due to the same clinical picture, and laboratory abnormalities detected in both diseases, and even similar histological features [10]. Moreover, low interobserver and intraobserver agreement rates were found among experienced liver transplant pathologists for the histopathologic differentiation of recurrent hepatitis C from ACR [3].

IFN-free direct-acting antiviral agents (DAAs) have improved tolerability and can potentially be used in posttransplant setting, which should result in better outcomes [11]. However, incorrect diagnosis may be detrimental, as failure to increase immunosuppression in patients with ACR may lead to acceleration of rejection, and inappropriate treatment of suspected acute rejection with high-dose pulse steroid therapy in misdiagnosed recurrent HCV can lead to aggravation of the disease, graft loss, and poor survival; hence, accurate diagnosis remains a critical issue [8, 12].

Macrophages hold a fundamental role in regulating inflammatory processes [13]. In particular, hepatic macrophages have the main role in the pathogenesis of acute and chronic liver injury through a wide range of different functions in the liver. The liver has about 80% of all body macrophages as local resident self-renewing macrophages, termed Kupffer cells. Blood monocytes can infiltrate into the liver; however, under steady-state conditions, blood monocyte-derived macrophages (BDM) do not contribute to the pool of local resident macrophages in the liver [14]. Previous work demonstrated that a reduction in number and function of circulating monocytes are strongly correlated with activation of systemic anti-inflammatory responses [15]. Macrophages in the liver can be distinguished based on their origin and certain marker expression. CD68 (cluster of differentiation 68) is a glycoprotein which binds low-density lipoprotein and is expressed on monocytes/macrophages [16]. Yang et al. [17] demonstrated that early activation of macrophages as a result of graft injury might play an important role in the accelerated ACR.

However, patients with HCV infection may have a significant increase in CD68+ expression in their portal tracts compared with normal tissue [18].

CXCL10 is well known in hepatitis C as a hepatocyte-derived chemotactic ligand and initiator of inflammatory cascades via its cognate receptor C-X-C motif receptor 3 (CXCR3). It is widely expressed on multiple cells of the innate immune system, including hepatic Kupffer cells, dendritic cells, natural killer (NK) cell, and neutrophils. Hence, these entire different innate immune cells are potential targets for CXCL10-mediated chemotaxis. [19]. Treatment with a CXCL11-neutralizing antibody reduced the number of CXCR3+ cells in the skin allograft and prolonged graft survival [20].

Till now, no precise marker for diagnosing ACR or recurrent HCV is currently available. This study aimed to determine the role of CD68, CD 11b, and CXCR3, as markers of resident Kupffer cells and BDM, in the differentiation between HCV reinfection and ACR in the postliver transplant setting using immunohistochemistry.

## 2. Methods

### 2.1. Specimens' Selection

This retrospective study was conducted on liver biopsies from 55 patients who had living donor liver transplantation (LDLT) for chronic HCV complications, whether cirrhosis and/or HCC, and who had developed elevated liver enzymes 6 months following transplantation. Twenty-nine patients developed recurrent HCV, and 26 patients were diagnosed as ACR based on histopathological examination. Laboratory investigation and histological criteria established the diagnosis which was confirmed by good response to treatment. Serological and clinical data were collected from the patients' files. The study was approved by the National Liver Institute Institutional Review Board.

Paraffin-embedded blocks of those liver biopsies were retrieved from the archive of the Pathology Department, National Liver Institute, Menoufia University, in the period between 2015 and 2017. Baseline characteristics including donor and recipient age and gender, pretransplantation HCC status, liver function tests, HCV-RNA level, and MELD score were determined.

### 2.2. Histopathological Evaluation

Serial liver sections in four micrometer thickness were cut from each paraffin-embedded block, for hematoxylin and eosin (H&E) staining and immunostaining. H&E staining was used for evaluation of histopathological changes including determination of the following parameters:

- Extent of infiltrate and the degree of portal inflammation identified by mononuclear infiltration of portal tracts
- Presence of interface hepatitis, spotty necrosis, confluent necrosis, steatosis, and cholestasis
- Presence or absence of fibrosis
- Presence of bile duct injury, venous endothelial injury, hepatic artery injury, or perivenular necrosis
- The nature and number of portal tract infiltrate: plasma cells, eosinophils, neutrophils, macrophages, and immunoblast cells

### 2.3. Immunohistochemistry

Immunohistochemistry was carried out for all tissues mentioned in the study. After deparaffinization and rehydration, hydrogen peroxide was applied to block nonspecific background staining. Heat-induced antigen retrieval was performed using citrate buffer solution low pH (pH 6) for CD11b and CX3CR1 antibodies and high pH (pH 9) for CD68. They are anti-human antibodies that arose in animals and recognize CD68 antigens on human macrophages [21]. Antigen retrieval solution was performed in a vegetable steamer for 20 minutes at 97°C followed by incubation for an additional 20 minutes in the warm buffer. All antibodies were incubated overnight at 4°C. Sections were incubated with a monoclonal mouse CD68 (clone KP1,0, DAKO A/S, Glostrup, Denmark, dilution 1 : 50), a rabbit polyclonal primary anti CD11 b (Novus Biologicals, Littleton, CO, USA, dilution 1 : 50), or a 1 : 200 dilution of a rabbit polyclonal primary anti-CX3CR1 antibody (Novus Biologicals, Littleton, CO, USA). Detection of the immunostaining was carried out utilizing the EnVisionTM FLEX/HRP detection system (DAKO A/S, Glostrup, Denmark) with the 3-diaminobenzidine (DAKO) as chromogen. After counterstaining with Mayer's hematoxylin, the slides were independently assessed by two pathologists for detection of each antibody.

Human lymph node (stains sinusoids) was positive tissue control for CD68; benign prostatic hyperplasia was positive tissue control for CD11 and human heart tissue for CX3CR1. Negative tissue controls were included in the protocol of staining by omitting the primary antibodies.

### 2.4. Interpretation of CD68, CD11b, and CXCR3

The immunoreactivity for CD68 was identified as membranous brownish discoloration of macrophages. The positive cells were quantified in three portal tracts and adjacent hepatic parenchyma per case (liver core). The positive cases were further divided according to the median number of CD68 positive cells into high expression (>40%) and low expression (<40%). The immunoreactivity for CD11b was assigned when cytoplasmic brownish discoloration was seen in mononuclear inflammatory cells. The positive cells were quantified in three portal tracts, interface, and adjacent hepatic parenchyma. The percentage of positivity was evaluated and expressed as range, mean, and median. The cases were divided into low expression when up to 30% of hepatocytes were positive and high expression when >30% of hepatocytes showed immunoreactivity. The immunoreactivity for CXCR3 was identified as cytoplasmic brownish discoloration of mononuclear inflammatory cells. The positive cells were quantified in three portal tracts and adjacent parenchyma per case (liver core). The positive cases were further divided according to the median number of CXCR3 positive cells into high expression (>20%) and low expression (<20%).

### 2.5. Statistical Analysis

Qualitative data was expressed in number and percentages, and quantitative data was expressed as mean and standard deviation. Fisher exact and chi-square tests were used to study the association between two qualitative variables. *t*-test was used for comparison between two quantitative variables. A *P* value of <0.05 was considered statistically significant.

## 3. Results

The baseline clinical, laboratory data of the studied patients are presented in Table 1. No significant difference was observed between the two groups regarding recipient age, gender, MELD score, and presence of HCC before transplantation (*P* = 0.11, 0.87, 0.57, and 0.54, resp.).

Histopathological features of recurrent chronic hepatitis C (CHC) and ACR are demonstrated in Table 2.

### 3.1. CD68 Expression

CD68 was detected in the inflammatory infiltrate in all cases of recurrent hepatitis C and ACR and was localized in portal tracts and adjacent parenchyma. The number of macrophages identified by CD68 immunostaining ranged from 10 to 60 in both groups. In recurrent hepatitis C, the mean ± SD was 32.3 ± 17.5 and a median of 30.0, while in cases of ACR, the mean ± SD was 37.7 ± 12.4 and a median of 40.0 without detectable significant difference between the two groups (*P* = 0.21) as shown in Table 3. Twelve cases (46.2%) of recurrent hepatitis C showed high expression of CD68 in comparison to 17 patients (65.4%) in ACR.

### 3.2. CD11b Expression

CD11b expression was detected among the mononuclear inflammatory infiltrate in portal areas, interface, or hepatic parenchyma. In recurrent hepatitis C, the number of macrophages identified by CD11b immunostaining ranged from 10 to 60; the mean ± SD was 26.5 ± 17.2 and the median was 30.0, while in ACR, ranging from 10 to 50, the mean ± SD was 17.3 **±** 12.5 and a median of 10.0, with a significant difference detected between the two groups (*P* = 0.03). High expression of CD11b (≥30%) was found in 14 (53.8%) and 5 cases (19.2%) of recurrent HCV and ACR, respectively (*P* = 0.01).

### 3.3. CXCR3 Expression

**Figure 1:**
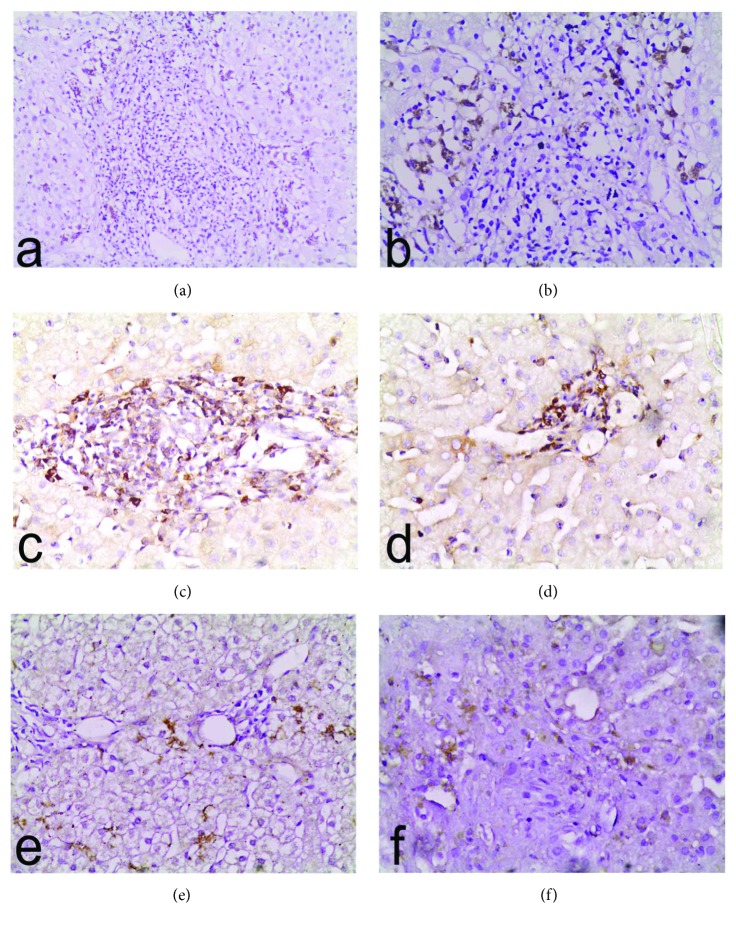
Immunohistochemistry of liver tissue postliver transplantation in recurrent hepatitis C (a, c, and e) and acute cellular rejection (b, d, and f) showing immunoreaction for CD68 (a and b), CD11b (c and d), and CXCR3 (e and f). Original magnification ×200.

CXCR3 expression was shown among the mononuclear inflammatory infiltrate in portal tracts, interface, or hepatic parenchyma. A significant difference between the two groups was detected (*P* < 0.001). The number of macrophages identified by CXCR3 immunostaining in recurrent hepatitis C ranged from 0 to 30; the mean ± SD was 6.2 **±** 8.5 and the median was 1.0. While in ACR, it ranged from 0 to 40; the mean ± SD was 20.4 **±** 10.4 and the median was 20.0. High expression of CXCR3 (≥20%) was found in 2 (7.7%) versus 18 (69.2%) cases of recurrent HCV and ACR, respectively (*P* < 0.001).  Figure 1 demonstrated immunohistochemical staining of CD68, CD11b, and CXCR3 in recurrent hepatitis C (Figures 1(a), 1(c), and 1(e)) and acute cellular rejection (Figures 1(b), 1(d), and 1(f)).

Subgroup analysis was performed according to presence or absence of HCC and revealed the same trend, although CD11b expression was not statistically significant between both groups (Tables 4 and 5).

Histological criteria established the diagnosis which was confirmed by good response to treatment in 94% of cases. Three cases were confusing and reassessment of the biopsy or even rebiopsy was mandatory. They are misdiagnosed as recurrent HCV posttransplantation, however, during follow-up, one patient was discovered to have lymphoproliferative malignancy and improved upon receiving systemic chemotherapy. The other two patients were found to have hyperacute and chronic rejection that unfortunately did not respond to increasing dose of immunosuppressive drugs and died. Another two patients diagnosed with ACR also died due to sepsis.

## 4. Discussion

Recurrent hepatitis C is characterized by the presence of lobular inflammation, apoptotic bodies, spotty necrosis, and lobular disarray, with portal lymphocyte predominance, while, in acute cellular rejection mixed portal/periportal inflammation composed of lymphocytes, plasma cells, and eosinophils, lymphocytic cholangitis and endothelialitis were observed. However, the histological diagnosis of HCV infection in the transplant setting may be altered, putting in mind that detectable serum HCV ribonucleic acid (RNA) after LT, even at a high level, does not necessarily indicate the presence of histologic recurrent hepatitis C [3]. Immunosuppression may alter the histological appearance of viral hepatitis posttransplant especially during the first few months; hence, classic histopathologic features of hepatitis C may be absent or modified. Moreover, ACR, ischemic injury, biliary obstruction, cytomegalovirus infection, or drug toxicity may superimpose chronic hepatitis [3]. Therefore, it is a challenge for the hepatopathologist to differentiate these overlapping microscopic features in some cases.

The present study aimed to determine the usefulness of macrophages' origin in the differential diagnosis of acute rejection and recurrent HCV after LT.

Macrophages have an important role in both recurrent hepatitis C and ACR posttransplantation. About 80% of all body macrophages reside in the liver and are furthermore patrolled by blood monocytes [22]. The circulating blood monocytes can principally infiltrate the liver and give rise to monocyte-derived macrophages, but this is characteristic to liver injury [23]. Liver macrophages have a wide range of functional heterogeneity; they may be pathogenic or even beneficial and they have been classified either into “proinflammatory” M1 or “immunoregulatory” M2 macrophages. Macrophages play a key role in acute and chronic liver inflammation and regression of liver disease. Upon injury to the liver, macrophages often perform immediate multiple functions including cytokine and chemokine secretion, leukocyte adhesion, phagocytosis, angiogenesis control, and extracellular matrix remodelling [24]. Although Kupffer cells can protect the transplanted liver, rejection of allografts was found also promoted by macrophages due to their antigen-presenting and cytokine-releasing function [24]. In cases of ACR, selective targeting and destruction of donor parenchymal cells occur through complement activation and the resultant membrane attack complex is responsible for lysis of the donor cells. Another pathway for the destruction of donor parenchymal cells in ACR is via antibody-dependent cellular cytotoxicity, involving other immune cell mediators, such as NK cells, macrophages (CD68-positive cells), and neutrophils [25].

CD68 has been proposed as an indicator for Kupffer cells [26] and is used to distinguish Kupffer cells from monocyte-derived macrophages [23, 27]. However, no single marker is currently able to definitely discriminate these populations. CD68 is a specific marker for the various cells of the macrophage lineage, including monocytes, Kupffer cells, histiocytes, giant cells, and osteoclasts. In this study, CD68 was expressed in all cases of recurrent hepatitis C and ACR, indicating the presence of macrophage infiltration of portal tracts in both groups. There was no significant difference in the number of macrophages highlighted by CD68 between cases of recurrent hepatitis C or ACR (*P* = 0.21). In accordance with this finding, CD68+ macrophages were found in the infiltrate of hepatic lobules in acute liver allograft rejection and the number of infiltrating cells correlated with the severity of the ACR in a previous report [28]. Also, the number of CD14+CD68+ Kupffer cells is increased in patients with viral hepatitis in another study [29]. In addition, CD68-positive monocytes were the main inflammatory cell-infiltrating renal graft in cases of ACR [30].

Monocyte-derived (freshly infiltrating) macrophages are characterized as CD11b^+^ F4/80^+^ cells by FACS in mice, whereas matured monocyte-derived and resident Kupffer cells are CD11b^lo^ F4/80^hi^ [31]. The number of CD11b(+), F4/80(+), CD11c(−), and CD206(+) (M2) macrophages in the liver of HCV transgenic mice was notably increased compared to control mice. These M2 macrophages in the liver produced elevated levels of IL-6 and TNF-*α*. These results suggested that inflammatory cytokines produced by M2-like macrophages contribute to the induction of chronic liver inflammation in HCV transgenic mice [32]. In agreement with these results, the present study demonstrated that the CD11b expression was in favor of recurrent hepatitis C compared with ACR (*P* = 0.03).

A massive necrosis of hepatocytes can provoke a strong inflammatory immune response within the liver [33] leading to secretion of diverse proinflammatory chemokines and cytokines, including interferon (IFN)-*γ* by liver-resident and -infiltrating immune cells [34] which perpetuate liver cell damage. IFN-*γ* strongly activates the transcription of the chemokines CXCL9, CXCL10, and CXCL11 [35, 36]. CXCR3, one of the peripheral blood monocyte surface markers, is the receptor for CXCL9, CXCL10, and CXCL11 chemokines which is expressed on various cell subpopulations within the liver, including liver endothelial cells, stellate cells, T cells, NK cells, and NKT cells [37, 38]. The interaction between these three chemokines and their receptor mediates the recruitment of T, NK, and NKT cells into the liver and their attachment to endothelial cells [39–41].

Our results clearly show great differences in CXCR3 expression between both groups. CXCR3 expression was significantly higher in ACR than recurrent HCV.

In conclusion, CD68 was expressed in both recurrent HCV infection and ACR. A significantly stronger CD11b deposits in liver biopsies of patients' suffering from recurrent HCV was detected. On the other hand, CXCR3 was a marker and plays a considerable role in acute rejection following liver transplantation suggesting the involvement of humoral mechanisms in ACR. Using immunohistochemistry beside clinical, laboratory, and histopathological criteria in discrimination between recurrent HCV and ACR may improve the diagnostic ability, morbidity, and mortality of these patients.

## Figures and Tables

**Table 1:** Demographic and laboratory characteristics of patients with recurrent chronic hepatitis C (CHC) and acute cellular rejection (ACR).

Parameters	Recurrent CHC (*n* = 29) %	ACR (*n* = 26)%
Recipient age (years)	47.9 ± 5.7	42.2 ± 13.4
Gender of recipient (M/F) *n*, %	24(82.7)/5(17.3)	26(100)/0(0)
Pretreatment HCC *n*, %	8 (27.6)	7 (26.9)
MELD score	15.8 ± 2.6	15.4 ± 2.0
Total bilirubin (mg/dL)	4.11 ± 5.0	5.1 ± 2.9
AST (IU/L)	127.1 ± 65.1	198.8 ± 164.1
ALT (IU/L)	165.9 ± 111.9	269.7 ± 257.6
GGT (IU/L)	721.7 ± 1041.4	1054.3 ± 1232.2
ALP (IU/L)	371.6 ± 211.3	400.9 ± 267
Serum albumin (gm/dL)	4.22 ± 0.33	3.97 ± 0.49

AST: aspartate aminotransferase; ALT: alanine aminotransferase; GGT: gamma glutamyl transferase; ALP: alkaline phosphatase.

**Table 2:** Histopathological characteristics of patients with recurrent chronic hepatitis C (CHC) and acute cellular rejection (ACR).

Parameters	Recurrent CHC (*n* = 29) %	ACR (*n* = 26) %
Extent of infiltrate *n*, %		
I	7 (24.13)	10 (38.5)
II	17 (58.62)	14 (53.8)
III	5 (17.24)	2 (7.7)
Spotty necrosis (absent/present) *n*, %	0 (0)/29 (100)	1 (3.8)/25 (96.2)
Confluent necrosis (absent/present) *n*, %	5 (17.24)/24 (82.76)	23 (88.5)/3 (11.5)
Perivenular necrosis (absent/present) *n*, %	20 (68.96)/9 (31.04)	8 (30.8)/18 (69.2)
Fibrosis (absent/present) *n*, %	9 (31.04)/20 (68.96)	18 (69.2)/8 (30.8)
Cholestasis (absent/present) *n*, %	23 (79.3)/6 (20.7)	17 (65.4)/9 (34.6)
Steatosis (absent/present) *n*, %	4 (13.8)/25 (86.2)	22 (84.6)/4 (15.4)
Bile duct injury (absent/present) *n*, %	16 (55.2)/13 (44.8)	3 (11.5)/23 (88.5)
Vascular injury (absent/present) *n*, %	28 (96.5)/1 (3.5)	4 (15.4)/22 (84.6)

**Table 3:** Comparison between recurrent chronic hepatitis C (CHC) and acute cellular rejection (ACR) regarding the studied markers (CD68, CD11b, and CXCR3).

Marker	Recurrent CHC	ACR	*t*-test	*P* value
CD68				0.21
Mean ± SD	32.3 ± 17.5	37.7 ± 12.4	−1.27	
Median	30.0	40.0		
CD11b				0.03
Mean ± SD	26.5 ± 17.2	17.3 ± 12.5	2.21
Median	30.0	10.0	
CXCR3				0.001
Mean ± SD	6.2 ± 8.5	20.4 ± 10.4	−5.40	
Median	10.0	20.0		

**Table 4:** Comparison between recurrent chronic hepatitis C (CHC) and acute cellular rejection (ACR) regarding the studied markers (CD68, CD11b, and CXCR3) in absence of HCC.

Marker (mean ± SD)	Recurrent CHC	ACR	*t*-test	*P* value
CD68	33.5 ± 17.3	36.7 ± 11.7	−0.59	0.56
CD11b	26.5 ± 15.8	18.6 ± 15.5	1.40	0.17
CXCR3	7.6 ± 9.7	18.0 ± 10.8	−2.85	0.008

**Table 5:** Comparison between recurrent chronic hepatitis C (CHC) and acute cellular rejection (ACR) regarding the studied markers (CD68, CD11b, and CXCR3) in presence of HCC.

Marker (mean ± SD)	Recurrent CHC	ACR	*t*-test	*P* value
CD68	30.0 ± 18.7	38.6 ± 15.7	−0.97	0.35
CD11b	26.6 ± 20.6	14.3 ± 5.3	1.53	0.15
CXCR3	3.3 ± 5.0	24.3 ± 11.3	−4.99	<0.001
